# Are all cancer survivors included when using electronically administered patient reported outcomes in primary healthcare cancer rehabilitation? A cross-sectional study

**DOI:** 10.1186/s41687-024-00753-5

**Published:** 2024-07-08

**Authors:** Sine Rossen, Mette Thønnings Sandager, Dorte Thoning Hofland, Claus Vinther Nielsen, Thomas Maribo

**Affiliations:** 1 Copenhagen Centre for Cancer and Health, Municipality of Copenhagen, Nørre Alle 45, Copenhagen N, DK-2200 Denmark; 2 Department of Public Health, Aarhus University, Aarhus, Denmark; 3 DEFACTUM, Central Denmark Region, Aarhus, Denmark; 4 Social Medicine and Rehabilitation, Region Hospital Goedstrup, Herning, Denmark

**Keywords:** Patient reported outcomes, PRO, Cancer rehabilitation, Inequality in health

## Abstract

**Background:**

Patient reported outcomes (PROs) are being used frequently in clinical practice. PROs often serve several purposes, such as increasing patient involvement, assessing health status, and monitoring and improving the quality-of-care at an aggregated level. However, the lack of representative PRO-data may have implications for all these purposes. This study aims to assess the association of non-administration of (not sending an electronic invite to PRO) and non-response to (not responding to PRO) electronically administered PROs with social inequality in a primary healthcare cancer rehabilitation setting. Furthermore, it examines whether the workflows surrounding PRO have an impact on non-administration and non-response.

**Methods:**

This is a cross sectional study using routinely collected data from electronic health records and registers including cancer survivors (CSs) over 18 years booked for an initial consultation in a primary healthcare cancer rehabilitation setting using PROs for systematic health status assessment. During the study period two different PRO platforms were used, each associated with different workflows. Non-administration and non-response rates were calculated for sociodemographic characteristics for each PRO platform. Crude and adjusted odds ratios were calculated using univariate and multivariate logistic regression.

**Results:**

In total, 1868 (platform 1) and 1446 (platform 2) CSCSs were booked for an initial consultation. Of these, 233 (12.5%) (platform 1) and 283 (19.6%) (platform 2) were not sent a PRO (non-administration). Among those who received a PRO, 157 (9.6%) on platform 1 and 140 (12.0%) on platform 2 did not respond (non-response). Non-administration of and non-response to PROs were significantly associated with lower socioeconomic status. Moreover, the workflows surrounding PROs seem to have an impact on non-inclusion in and non-response to PROs.

**Conclusions:**

Non-administration of and non-response to PROs in clinical practice is associated with determinants of social inequality. Clinical workflows and the PRO platforms used may potentially worsen this inequality. It is important to consider these implications when using PROs at both the individual and aggregated levels. A key aspect of implementing PROs in clinical practice is the ongoing focus on representativeness, including a focus on monitoring PRO administration and response.

**Supplementary Information:**

The online version contains supplementary material available at 10.1186/s41687-024-00753-5.

## Background

Patient reported outcome (PRO) questionnaires are increasingly used in routine clinical practice for long term conditions, including cancer [1–4]. On the individual level, PROs have the potential to increase patient-centred care by providing the patient’s perspective and facilitating dialogue in consultations by identifying and focusing on the patient’s current health status [5–11]. On the aggregated level, PRO-data may be used for quality monitoring and improvement in clinical practice as well as research purposes [12, 13]. In the field of oncology, most studies on the use of PROs have been performed in hospital settings in relation to patients undergoing active treatment, whereas knowledge about routine use in other settings is scarce [2].

One challenge of using PRO questionnaires in clinical practice is the potential to exacerbate existing health inequalities. The integration of PROs into routine clinical care may face challenges arising from a variety of factors concerning both patients and healthcare professionals [9, 14, 15]. The organization of PRO data collection, including aspects such as incorporation into existing clinical workflows or the design of the PRO collection system, can impact patient participation in PRO assessments [3, 16]. Studies have shown that non-response is higher in men, persons with higher ages, ethnic minorities, individuals with lower socioeconomic status, and persons living alone [17–21]. The same pattern is seen for the use of electronically administered PROs [20]. Lower socioeconomic status has been associated with lower health literacy, or eHealth literacy [22, 23], and the use of PROs or electronic PROs may exclude populations not able to fill out or finish questionnaires due to low health literacy. Also, cognitive impairments or language barriers may hinder participation in PROs [24, 25]. A lack of representative PRO data not only excludes some populations from the individual benefits of using PROs but also introduces bias in the data used to evaluate clinical care and thus a risk of making decisions about health service innovations that only benefit certain populations. It is therefore important that healthcare organisations have awareness of the availability and accessibility of services to accommodate patients with different strengths and limitations, i.e., organisational health literacy responsiveness [26, 27]. Most studies report on response rates for PRO, whereas studies focusing on administration or distribution rates are few [28].

The objective of this study was to assess whether non-administration of (i.e., not sending an invite to PRO) and non-response to (i.e., not responding to PRO) electronically administered PROs were associated with social inequality in health among cancer survivors referred to rehabilitation in primary healthcare cancer rehabilitation. Additionally, the study aimed to determine whether the workflows surrounding PRO and the PRO platform impacted non-administration and non-response.

## Methods

### Study design

The study is cross sectional using routinely collected data from electronic health records.

### Study setting

In Denmark, cancer rehabilitation is provided by municipalities in primary healthcare settings at a community level [29]. In the municipality of Copenhagen, which covers approximately 650,000 inhabitants, cancer rehabilitation is handled by the Copenhagen Centre for Cancer and Health. Rehabilitation is focussed on reaching or maintaining optimal quality of life by interventions aimed at physical, social, psychological, and cognitive function. Approximately 1200 cancer survivors (CSs) are referred yearly to the centre from hospitals (approximately 95%) or general practitioners. At the time of referral, around 58% of CSs have been diagnosed with cancer within the last 1–3 months, 24% within 4–11 months, and 18% more than a year ago. This means that many CSs are undergoing active treatment while participating in rehabilitation. Therefore, an important task for the healthcare professionals in the centre (nurses, physiotherapists, dieticians, or occupational therapists) is the assessment of the changing health status. In 2019, the centre implemented the use of electronically administered PRO in connection with the initial consultation as a tool to support systematic assessment of health status and thereby an indication of the rehabilitation needs the CS might have, to support the dialogue between the healthcare professional and CS, and for quality improvement purposes. In the present setting, it is standard practice for healthcare professionals to actively incorporate PRO into the dialogue. This emphasis on PRO utilisation has been a major focus of the management within the centre. Healthcare professionals are trained how to use the PRO platform by an internal process consultant who also facilitates exchange of experiences among the healthcare professionals about the active use of PRO during the consultation. Considering that most CSs are recently diagnosed, many may undergo emotional distress or find themselves in the initial phases of treatment, frequently encountering accompanying side effects. PRO thus serves as a snapshot of the health status of CSs who are navigating through a highly changeable period in their lives, and the relevance of their responses may vary at the time of the consultation. Therefore, a flexible approach to the active use of PRO is utilised, where, depending on the circumstances, responses may be addressed over shorter or longer periods during the consultation. The PRO used comprises 62 questions. It is a combination of validated scales and items designed specifically for health status assessment at the initial consultation. The core of the questionnaire is the 27-item Functional Assessment of Cancer Therapy-General (FACT-G) questionnaire, which measures health-related quality of life status and covers physical, social, emotional, and functional well-being [30]. These items cannot be skipped when filling out PRO. Other items cover lifestyle factors (smoking, alcohol, diet, exercise), self-rated health and cancer distress (distress thermometer), family situation, occupational situation, disease, as well as priorities and concerns. These items can be skipped when filling out PRO. On average, it takes between 10 and 20 min to answer PRO.

### Workflows surrounding PRO and PRO platforms

**Fig. 1:**
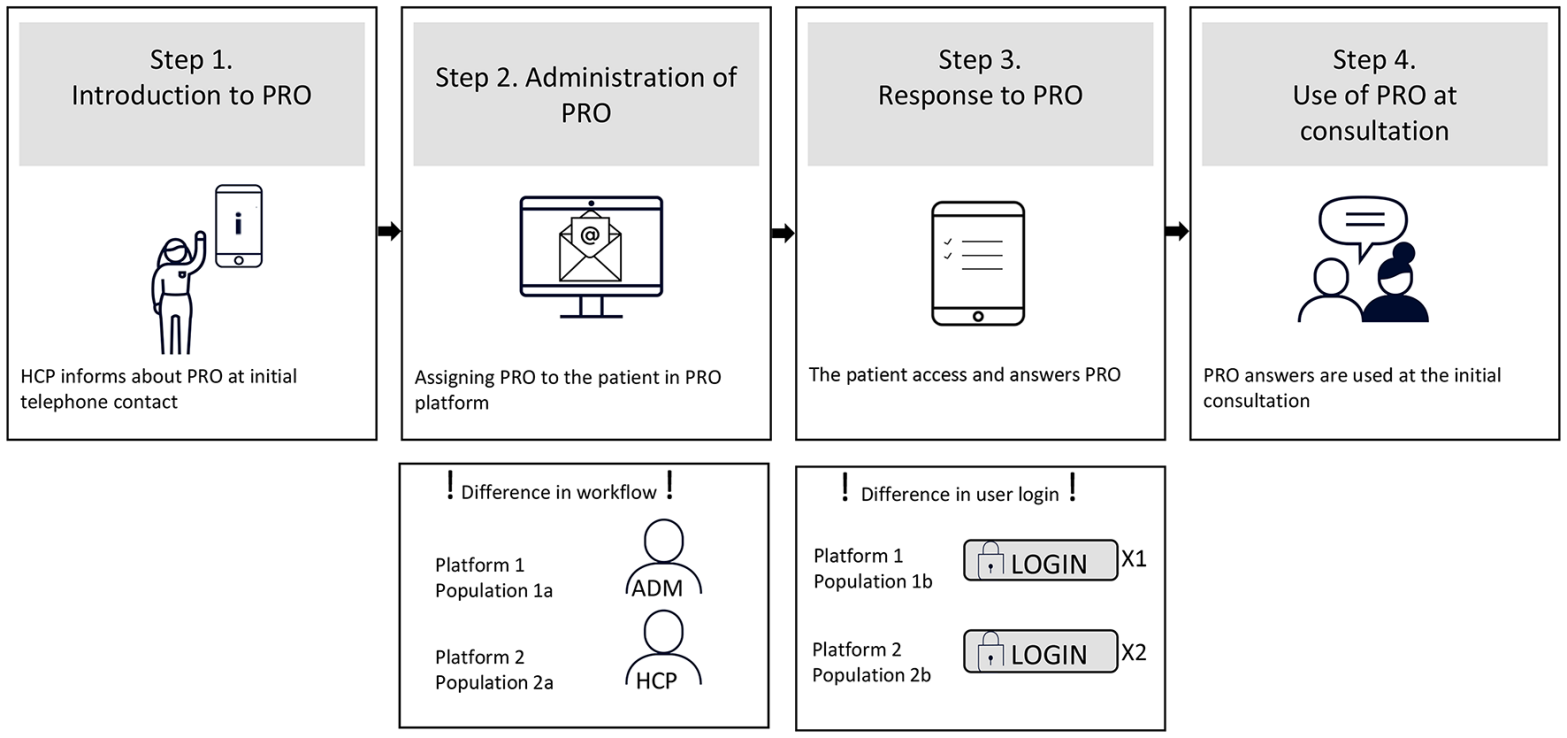
Workflow in connection with PRO and differences between the two web-based questionnaire platforms. *ADM* administrative staff, *HCP* healthcare professional, *PRO* patient reported outcome

Two different web-based PRO questionnaire platforms were used during the study period. Platform 1, a commercially available online survey platform, was used from April 2019 to February 2021. Platform 2, an online platform developed specifically for PRO use in the rehabilitation centre, was used from March 2021-August 2022. None of the platforms were integrated with the electronic health record. The workflow for PRO can be divided into four overarching steps. For both staff and patients, there are differences in several of these steps between the two platforms (Fig. 1).

Step 1: For both platforms, referred CSs were contacted by phone by a healthcare professional, and an initial consultation was booked. The CS was informed about PRO, emphasising its importance as preparation for the consultation, and that answers would be used during the consultation to help focus on the individual’s health status and accompanying needs for rehabilitation (Step 1, Fig. 1). If, during the phone call, it became evident that the CS was exempted from the secure national online digital mailbox or had difficulties accessing digital PRO, the healthcare professional suggested the possibility of answering PRO on a tablet in the centre just prior to the initial consultation.

Step 2: For platform 1, the healthcare professional informed the administration about the booked consultation, and administrative staff assigned PRO to the CS. For platform 2, the healthcare professional assigned PRO to the CS (Step 2, Fig. 1). Approximately one week prior to the booked consultation, a link to PRO was electronically sent via the secure national online digital mailbox used for communication between individuals and the public sector. The electronic mailbox is linked to a person’s civil registration number and is accessible using a digital ID authenticator app, code display or chip. For platform 1, administrative staff checked PRO response status daily for consultations the following day and sent reminders via the platform to those who had not answered. For platform 2, automatic reminders were sent if there was no answer one day prior to the consultation.

Step 3: For platform 1, a personalised link was generated. The CS logged in to their secure digital mailbox with their digital ID to access the link and was guided directly to the PRO. For Platform 2, data security was increased, and a general link was used, so the CS had to log in to the secure digital mailbox with their digital ID, access the link, and use their digital ID again to access the PRO (Step 3, Fig. 1). CSs completed the PRO at home using a smartphone, tablet, or computer. During completion, some questions were optional, but the questions from FACT-G were mandatory. CSs exempted from the national secure digital mailbox or those who found it too difficult to access had the possibility of completing the PRO on a tablet in the centre immediately prior to the consultation. In the region, the proportion of inhabitants exempted from the national digital mailbox has decreased from 6.4% in 2019 to 4.9% in 2022 [31].

Step 4: Answers to PRO were discussed with the CS at the consultation (Fig. 1, Step 4). For platform 1, administrative staff downloaded PRO answers for upcoming initial consultations, copied them into an excel sheet with colour-coding, and uploaded the excel sheet to the electronic health record so the healthcare professional could prepare for the consultation. Platform 2 was developed specifically for PRO use in the rehabilitation centre, aiming to facilitate healthcare communication around PRO in Step 4, Fig. 1. This was achieved by providing healthcare professionals with an easy overview of PRO responses for upcoming consultations, automatically generated colour-coded PRO reports, and the ability to observe changes in PRO answers over time. For both platforms, the colour coding serves as a visual tool to quickly convey the health status of the CSs based on their responses. Responses indicating severe symptoms or distress are highlighted in red, while those indicating mild or no symptoms are green.

### Participants

**Fig. 2:**
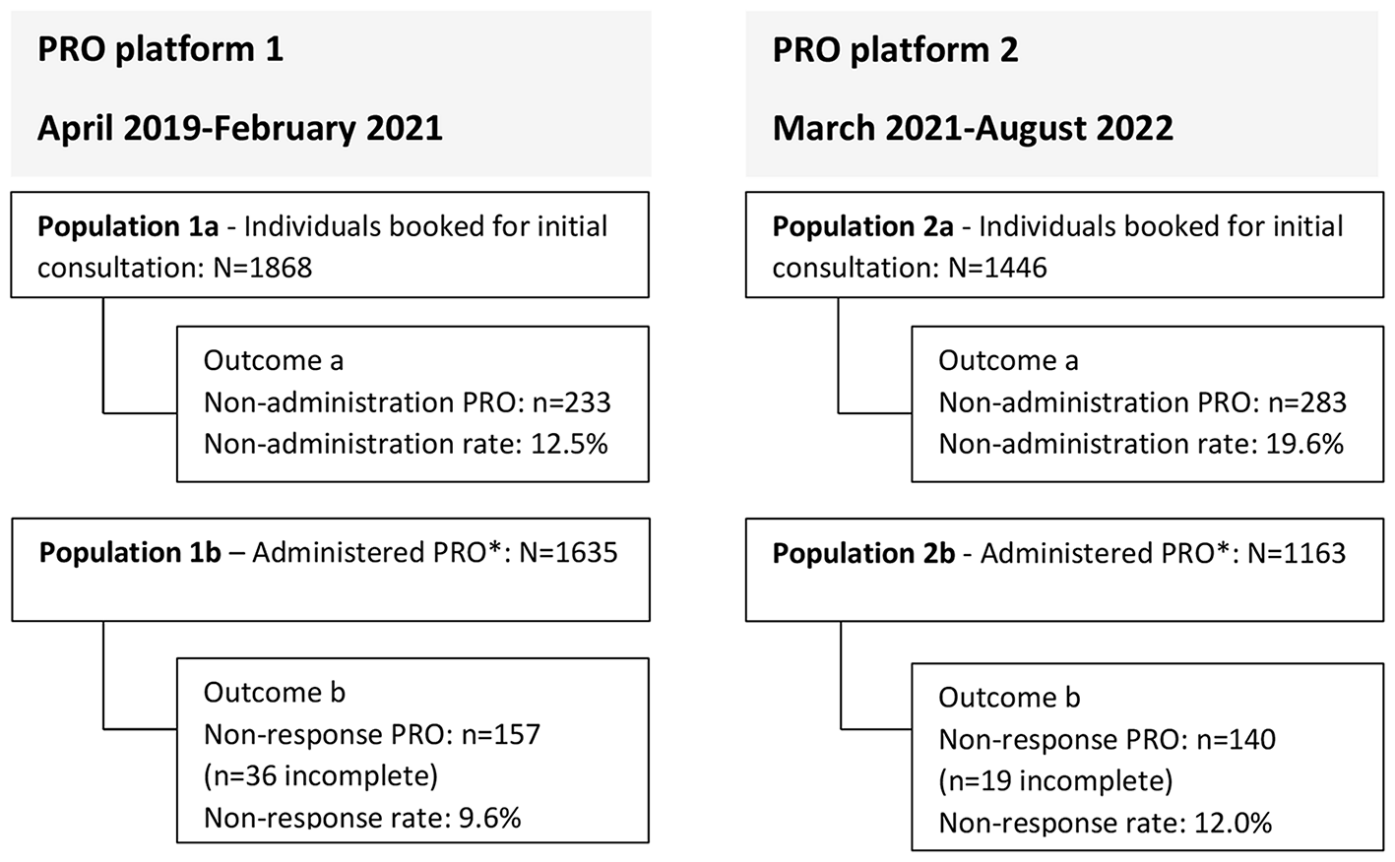
Patient flow for the two PRO platforms. *Including those administered in the centre prior to the initial consultation. *PRO* patient reported outcome

We identified CSs over 18 years referred to cancer rehabilitation and booked for an initial consultation at the Copenhagen Centre for Cancer and Health from 1 April 2019 to 31 August 2022. If a CS had been referred and booked for a consultation more than once in the study period, the first referral was used in this study. Identified CSs were divided into two groups according to the PRO platform used at the time of referral. For each PRO platform, two populations were formed: those booked for an initial consultation (population 1a and 2a) and those who had been sent a PRO (population 1b and 2b) (Fig. 2).

### Data collection

The outcomes under study were non-administration for populations 1a and 2a and non-response for populations 1b and 2b. Non-administration was defined when the CS was booked for an initial consultation, but was not sent a link to PRO. Non-response was defined when the CS had been sent a link to PRO but had not answered PRO. A response was considered valid if the CS had answered the 27 mandatory items of FACT-G. Age, sex, and educational level were selected as covariables as they have previously been shown to have an impact on the response rate [17–20]. Also, educational level is a highly contributing and robust marker for social inequality in cancer, as disease may affect other markers such as occupational status and income [32].

Data on age at referral, sex, diagnosis, administration of the PRO, and response to the PRO were retrieved from the electronic health record and the platforms used for administering the PRO. Additional sociodemographic data on individuals included in the study were obtained from registers at Statistics Denmark. The highest attained level of education was categorised according to the International Standard Classification of Education (ISCED) as low (ISCED 0–2), medium (ISCED 3–4), and high (ISCED 5–8) [33, 34]. The disposable family income level was divided in tertiles, high (~42,850–102,900€), medium (~26,800–42,800€), low (~9,000–26,750€), for each population in the study [35]. In 2020, the median disposable income level in Denmark was ~34,100€ [36]. Occupational status was categorised into employment, retirement pension (including early retirement), and social benefits (unemployment, sick leave, social security, disability pension, and education grant) [37]. Cohabitation status was categorised as ’living alone: yes’ and ’living alone: no’ [38]. Ethnicity was categorised into Danish origin, Western origin, and non-western origin, based on country of origin and parents birth or citizenship country [39, 40].

Healthcare professionals were encouraged to document it in the electronic health record if, during the telephone conversation (Step 1, Fig. 1), CSs expressed that they did not wish to receive PRO or if the healthcare professional assessed that the CS would be unable to complete PRO due to, e.g., language or cognitive barriers. To gain further insight into non-administration, an audit of the health records of those who had not received PRO was conducted. Reasons for non-administration of PRO were recorded and subsequently categorised into themes.

### Statistical analysis

Sociodemographic characteristics are presented as numbers and proportions. Audit results are presented as numbers. Associations between PRO platform and non-administration rates and non-response rates in PRO were analysed by crude and multivariate logistic regression adjusting for age, sex (male or female), and education (high, medium, low). A confidence interval of 95% was used.

For each PRO platform, non-administration rates, non-response rates, and crude odds ratios were determined for each sociodemographic characteristic using logistic regression. Multivariate logistic regression adjusting for age, sex, and education was used to estimate adjusted odds ratios. The number of CSs in the individual analyses varied due to missing values for variables obtained via registers. Statistical analyses were performed using R.

## Results

### Participants

During the study period, 1868 (platform 1) and 1446 (platform 2) CSs were booked for an initial consultation (population 1a and 2a, respectively). Of these, 1635 (87.5%) and 1163 (80.4%) were included in PRO (population 1b and 2b, respectively) (Fig. 2). The sociodemographic characteristics of the two populations can be seen in Table 1. Across populations, most CSs were female, aged 40–64 years, had a high level of education, and were of Danish origin. The most predominant diagnoses were cancers of the breast, digestive organs, and respiratory and intrathoracic organs (Supplementary table).

**Table 1:** Sociodemographic characteristics of CSs booked for an initial consultation (populations 1a and 2a) and CSs administered PRO (populations 1b and 2b) for the PRO platforms

	Booked for initial consultation, n (%)		Administered PRO, n (%)	
	Platform 1, population 1a	Platform 2, population 2a	Platform 1, population 1b	Platform 2, population 2b
n	1868	1446	1635	1163
Sex				
Female	1204 (64.5)	912 (63.1)	1072 (65.6)	734 (63.1)
Male	664 (35.5)	534 (36.9)	563 (34.4)	429 (36.9)
Age				
18–39 years	190 (10.2)	128 (8.9)	171 (10.5)	112 (9.6)
40–64 years	887 (47.5)	683 (47.2)	792 (48.4)	590 (50.7)
65–74 years	524 (28.1)	395 (27.3)	452 (27.6)	310 (26.7)
≥75 years	267 (14.3)	240 (16.6)	220 (13.5)	151 (13.0)
Educational level				
High	811 (43.4)	664 (45.9)	747 (45.7)	579 (49.8)
Medium	640 (34.3)	499 (34.5)	566 (34.6)	403 (34.7)
Low	342 (18.3)	234 (16.2)	271 (16.6)	155 (13.3)
Missing, n	75 (4.0)	49 (3.4)	51 (3.1)	26 (2.2)
Family income level				
High	584 (31.3)	473 (32.7)	515 (31.5)	381 (32.8)
Medium	584 (31.3)	473 (32.7)	516 (31.6)	382 (32.8)
Low	585 (31.3)	473 (32.7)	515 (31.5)	382 (32.8)
Missing, n	115 (6.2)	27 (1,9)	89 (5.4)	18 (1.5)
Occupational status				
Employment	743 (39.8)	635 (43.9)	691 (42.3)	573 (49.3)
Age-related pension	735 (39.3)	564 (39.0)	620 (37.9)	409 (35.2)
Social benefits	387 (20.7)	245 (16.9)	323 (19.8)	181 (15.6)
Missing, n	3 (0.2)	2 (0.1)	1 (0.1)	0 (0.0)
Ethnicity				
Danish origin	1536 (82.2)	1203 (83.2)	1379 (84.3)	1003 (86.2)
Western origin	126 (6.7)	92 (6.4)	108 (6.6)	62 (5.3)
Non-western origin	161 (8.6)	123 (8.5)	120 (7.3)	82 (7.1)
Missing, n	45 (2.4)	28 (1.9)	28 (1.7)	16 (1.4)
Living alone				
No	864 (46.3)	657 (45.4)	770 (47.1)	543 (46.7)
Yes	927 (49.6)	745 (51.5)	808 (49.4)	596 (51.2)
Missing, n	77 (4.1)	44 (3.0)	57 (3.5)	24 (2.1)

### Non-administration

The non-administration rate increased from 12.5% to 19.6% with the organizational change from PRO platform 1 to 2 with non-administration being significantly more likely for platform 2 (Table 2). For both platforms, non-administration was significantly associated with lower educational levels, lower family income levels, social benefits, and being of non-western origin (Table 3). Adjusting for covariables lowered the odds ratio, but associations remained significant. For platform 1, an age of ≥75 years was significantly associated with non-administration but was no longer significant when adjusting for sex and educational level. For platform 2, ages 65–74 years and ≥75 years were significantly associated with non-administration. Adjusting for covariables increased the point estimates and exhibited odds of 2.22 to 4.58-fold respectively, for non-administration. Living alone was not associated with non-administration for either platform. An audit of reasons for non-administration documented in the health record showed that no reason had been recorded for 73 out of 233 CSs and 94 out of 283 CSs for platforms 1 and 2, respectively. The major reason for non-administration was that rehabilitation was not initiated. All CSs were contacted by phone and despite referral, rehabilitation was not initiated; therefore, no PRO was administered. Other reasons included CSs’ inability to understand Danish, technical issues such as lacking a national digital ID or IT equipment, CS reluctance to fill out PRO at the centre, CS opting out of PRO, or factors such as cognitive issues, age, impaired vision, or psychological concerns (Table 4).

**Table 2:** Associations between the organisation of PRO and participation in PRO

	Non-administration			Non-response		
	n (%)	Crude OR (95%CI)	Adjusted* OR (95%CI)	n (%)	Crude OR (95%CI)	Adjusted* OR (95%CI)
Platform 1	233 (12.5)	Reference	Reference	157 (9.6)	Reference	Reference
Platform 2	283 (19.6)	1.71 (1.41; 2.06)	1.77 (1.44; 2.17)	140 (12.0)	1.29 (1.01; 1.64)	1.41 (1.10; 1.81)

*OR* Odds ratio, *CI* confidence interval

*Adjusted for sex, age, and educational level

**Table 3:** Associations between sociodemographic factors and non-administration for the two PRO platforms

	PRO platform 1			PRO platform 2		
	Non-administration, n (%)	Crude OR (95%CI)	Adjusted* OR (95%CI)	Non-administration, n (%)	Crude OR (95%CI)	Adjusted* OR (95%CI)
Sex**						
Female	132 (11.0)	Reference	Reference	178 (19.5)	Reference	Reference
Male	101 (15.2)	1.46 (1.10; 1.92)	1.25 (0.93; 1.69)	105 (19.7)	1.01 (0.77; 1.32)	0.92 (0.68; 1.23)
Age**						
18–39 years	19 (10.0)	Reference	Reference	16 (12.5)	Reference	Reference
40–64 years	95 (10.7)	1.08 (0.66; 1.86)	1.02 (0.59; 1.89)	93 (13.6)	1.10 (0.64; 2.01)	1.24 (0.67; 2.48)
65–74 years	72 (13.7)	1.43 (0.86; 2.51)	1.33 (0.75; 2.50)	85 (21.5)	1.92 (1.11; 3.52)	2.22 (1.19; 4.45)
≥75 years	47 (17.6)	1.92 (1.10; 3.47)	1.69 (0.91; 3.30)	89 (37.1)	4.13 (2.35; 7.64)	4.58 (2.44; 9.27)
Educational level**						
High	64 (7.9)	reference	Reference	85 (12.8)	Reference	Reference
Medium	74 (11.6)	1.53 (1.07; 2.17)	1.40 (0.98; 2.01)	96 (19.2)	1.62 (1.18; 2.23)	1.41 (1.01; 1.96)
Low	71 (20.8)	3,06 (2.12; 4.41)	2.79 (1.92; 4.06)	79 (33.8)	3.47 (2.44; 4.95)	2.87 (1.98; 4.14)
Family income level						
High	35 (6.0)	Reference	Reference	44 (9.3)	Reference	Reference
Medium	72 (12.3)	2.21 (1.46; 3.40)	1.89 (1.23; 2.97)	82 (17.3)	2.04 (1.39; 3.04)	1.79 (1.19; 2.72)
Low	100 (17.1)	3.23 (2.18; 4.90)	2.42 (1.57; 3.80)	148 (31.3)	4.44 (3.10; 6.46)	3.10 (2.07; 4.71)
Occupational status						
Employment	52 (7.0)	Reference	Reference	62 (9.81)	Reference	Reference
Age-related pension	115 (15.6)	2.46 (1.76; 3.50)	2.23 (1.00; 5.40)	155 (27.5)	3.50 (2.55; 4.85)	1.19 (0.65; 2.27)
Social benefits	64 (16.5)	2.63 (1.79; 3.90)	2.17 (1.41; 3.36)	64 (26.1)	3.27 (2.22; 4.82)	2.90 (1.90; 4.45)
Ethnicity						
Danish origin	157 (10.2)	Reference	Reference	200 (16.6)	Reference	Reference
Western origin	18 (14.3)	1.46 (0.84; 2.42)	1.89 (1.07; 3.17)	30 (32.6)	2.43 (1.51; 3.82)	3.24 (1.92; 5.37)
Non-western origin	41 (25.5)	3.00 (2.01; 4.41)	2.86 (1.85; 4.35)	41 (33.3)	2.51 (1.66; 3.74)	3.03 (1.92; 4.73)
Living alone						
No	94 (10.9)	Reference	Reference	114 (17.4)	Reference	Reference
Yes	119 (12.8)	1.21 (0.91; 1.61)	1.12 (0.82; 1.52)	149 (20.0)	1.19 (0.91; 1.56)	0.95 (0.70; 1.28)

*OR* Odds ratio, *CI* confidence interval

*Adjusted for sex, age, and educational level

**Sex, age, and educational level were adjusted for the two other covariables respectively

**Table 4:** Reasons for non-administration of PRO

	Platform 1 (n = 233)	Platform 2 (n = 283)
No reason documented in the health record	73	94
No rehabilitation initiated*	81	83
Technical issues**	25	38
Non-Danish speaking	25	22
Impaired vision/cognitive deficits/ dyslexia/age	10	9
CS opting out of PRO	9	6
Emotional distress/lack of energy	5	9
CS does not want to complete PRO in the centre	0	6
Other***	2	10

Numbers do not sum to total n as several reasons may apply to the same CS, such as both non-Danish speaking and emotional distress

*CS* cancer survivor

*The CS has changed their mind on wanting rehabilitation, are terminal or are referred with a specific problem that only requires one service (e.g., one manual physiotherapeutic treatment)

**CS is exempted from the national digital mailbox or lack technical equipment (e.g. computer/tablet/smartphone)

***e.g., healthcare professional forgot to administer PRO or PRO declined, but no specific reason was stated, and it was not clear if it was the CSs or the healthcare professional’s choice

### Non-response

The non-response rate increased from 9.6% to 12.0% with the organisational change from PRO platform 1 to 2 (Table 2). Non-response was significantly more likely for platform 2. For each platform, non-response was significantly associated with lower educational levels, lower disposable family income levels, and retirement pension, or social benefits (Table 5). This was also the case when adjusting for covariables except for retirement pension. Higher age was associated with non-response for both platforms but only remained significant for the highest age group for platform 2 after adjustment. For platform 1, living alone was significantly associated with non-response. For platform 2, being of non-western origin was significantly associated with non-response.

**Table 5:** Associations between sociodemographic factors and non-response for the two PRO platforms

	PRO platform 1			PRO platform 2		
	Non-response, n (%)	Crude OR (95%CI)	Adjusted* OR (95%CI)	Non-response, n (%)	Crude OR (95%CI)	Adjusted* OR (95%CI)
Sex**						
Female	99 (9.2)	Reference	Reference	86 (11.7)	Reference	Reference
Male	58 (10.3)	1.13 (0.80; 1.58)	1.01 (0.70; 1.44)	54 (12.6)	1.08 (0.75; 1.56)	1.02 (0.70; 1.48)
Age**						
18–39 years	10 (5.8)	Reference	Reference	12 (10.7)	Reference	Reference
40–64 years	71 (9.0)	1.59 (0.84; 3.33)	1.40 (0.71; 3.08)	61 (10.3)	0.96 (0.52; 1.93)	1.16 (0.60; 2.49)
65–74 years	52 (11.5)	2.09 (1.08; 4.46)	1.75 (0.87; 3.93)	36 (11.6)	1.09 (0.56; 2.27)	1.20 (0.60; 2.66)
≥75 years	24 (10.9)	1.97 (0.94; 4.43)	1.66 (0.75; 3.95)	31 (20.5)	2.15 (1.08; 4.56)	2.16 (1.03; 4.88)
Educational level**						
High	46 (6.2)	Reference	Reference	46 (7.9)	Reference	Reference
Medium	46 (8.1)	1.35 (0.88; 2.06)	1.30 (0.84; 2.00)	63 (15.6)	2.15 (1.44; 3.23)	2.03 (1.35; 3.07)
Low	56 (20.7)	3.97 (2.61; 6.06)	3.73 (2.44; 5.74)	28 (18.1)	2.55 (1.52; 4.22)	2.38 (1.41; 3.97)
Family income level						
High	16 (3.1)	Reference	Reference	26 (6.8)	Reference	Reference
Medium	70 (13.6)	4.11 (2.39; 7.48)	3.65 (2.09; 6.72)	41 (10.7)	1.64 (0.99; 2.77)	1.58 (0.94; 2.71)
Low	60 (11.6)	4.89 (2.88; 8.84)	3.50 (1.98; 6.52)	71 (18.9)	3.12 (1.96; 5.09)	2.69 (1.62; 4.58)
Occupational status						
Employment	40 (5.8)	Reference	Reference	47 (8.2)	Reference	Reference
Age-related pension	69 (11.1)	2.04 (1.37; 3.08)	0.82 (0.38; 1.93)	59 (14.4)	1.89 (1.26; 2.84)	1.09 (0.49; 2.56)
Social benefits	48 (14.9)	2.84 (1.83; 4.44)	2.09 (1.28; 3.41)	34 (18.8)	2.59 (1.60; 4.16)	2.33 (1.40; 3.84)
Ethnicity						
Danish origin	122 (8.8)	Reference	Reference	107 (10.7)	Reference	Reference
Western origin	14 (13.0)	1.53 (0.82; 2.69)	1.92 (0.98; 3.50)	9 (14.5)	1.42 (0.64; 2.83)	1.28 (0.54; 2.66)
Non-western origin	17 (14.2)	1.70 (0.96; 2.86)	1.71 (0.94; 2.96)	22 (26.8)	3.07 (1.78; 5.14)	3.07 (1.73; 5.29)
Living alone						
No	57 (7.4)	Reference	Reference	63 (11.6)	Reference	Reference
Yes	92 (11.4)	1.61 (1.14; 2.28)	1.47 (1.02; 2.12)	72 (12.1)	1.05 (0.73; 1.50)	0.97 (0.67; 1.42)

*OR* Odds ratio, *CI* confidence interval

*Adjusted for sex, age, and educational level

**Sex, age, and educational level were adjusted for the two other covariables respectively

## Discussion

This study demonstrates that non-administration of and non-response to PROs were associated with lower socioeconomic status. Furthermore, the study found that workflows and platforms used had an impact on non-administration of and non-response to PROs. An altered workflow in connection with the implementation of platform 2 resulted in an increase in non-administration. Platform 1 was replaced by platform 2 to support the healthcare professional-CS dialogue with a better visualisation of PRO answers, comply with regulations for health records, general data protection rights (GDPR), and to increase data security. A side-effect of the increased security might have been the statistically significant increase in inequality in who responded to PROs. However, this must be balanced against the benefits in terms of improved usefulness in the consultation (e.g., automatic colour-coding, changes in responses over time) and safe storage of health record data. Although platform 1 is no longer in use, the insights gained regarding the influence of various workflows and platforms on administration and response in clinical practice are transferable. The results can inform the implementation of PRO platforms and the development of effective workflow strategies around them.

### Non-administration

Most studies report on response rates in connection with the use of PRO in clinical studies with defined inclusion criteria, whereas few studies focus on the administration of PROs to patients in a pragmatic setting. In the current study, non-administration of PRO ranged from 9.6% to12.0%. In comparison, an out-patient clinic that utilizes both PRO-based and conventional follow-up determines inclusion in PRO-based follow-up through healthcare professionals’ assessment and the patients’ preferences. This clinic reports that approximately 50% of patients are included in PRO-based follow-up [41]. Another study in an outpatient clinic found administration rates of approximately 90 to95% after implementation of PROs via unmounted tablets for data collection in the waiting room immediately after patient registration [42]. In line with our findings, these studies found that the administration of PRO was significantly lower for older patients [42] and socioeconomically disadvantaged patients [41].

We found an increase in non-administration with the organisational shift from platform 1 to platform 2. This may be due to the shift in workflow that created additional demands on healthcare professionals. For example, the PRO platform was not integrated with the electronic health record, and healthcare professionals had to log in to a different platform to assign PRO to CSs. Other studies have shown that healthcare professionals’ competing clinical priorities may threaten the inclusion in PRO [15, 43, 44]. We found that, in contrast to platform 1, people over 65 years old were less likely to be included with the change to platform 2. It is possible that CSs who were not administered PROs in the current setting may also be less inclined to respond to them. The characteristics of this subpopulation were associated lower socioeconomic status which has previously been related to non-response [19, 24]. The intention was to encompass all CSs scheduled for an initial consultation in the PRO, which unfortunately was not accomplished for any of the platforms. An audit of patient records revealed that healthcare professionals recorded various reasons for non-administration. The major reason was that no rehabilitation was initiated. This is a valid reason for non-administration of PRO and indicates that the true non-administration rate may be lower. Factors such as technical barriers, language barriers, cognitive issues, age, impaired vision, or psychological concerns were also recorded. However, it was not always clear whether it was a healthcare professional assessment or the CSs choice to opt out of PRO. In palliative care, gatekeeping, where healthcare professionals shield their patients from entering studies or using PROs because of a notion that it will be too burdensome for the patient to participate due to, e.g., disease burden or cognitive impairment, is a known concern [45, 46]. Similarly, in a study implementing a digital version of the guided self-determination tool, the healthcare professionals had concerns on the patients’ behalf about age, digital skills, and cognitive function [47].

### Non-response

For both platforms, non-response was associated with indicators of lower socioeconomic status such as educational level, family income level, and occupational status. This is in line with findings from other studies [17–21]. In the current study, electronic PRO platforms were used. Some studies have shown that response rates were higher for manual PROs compared to electronic PRO platforms [17, 18, 21], while others have found only minor differences [48, 49]. The use of electronic PRO platforms may be an issue for persons of higher age, lower educational level, or ethnic minorities [17], although a Danish study showed only minor socioeconomic differences between non-respondents of paper- or web-based PROs [21]. This study allows us to compare non-response between two different electronic platforms. Notably, the non-response rate increased from 12.5% for platform 1 to 19.6% for platform 2. This can possibly be attributed to the increased complexity for CSs using platform 2, which was designed to comply with regulations for patient records. In this case, CSs had to first log on to their personal digital mailbox to access the link to the platform and then use their digital ID to access the PRO platform. Interestingly, for platform 2, we observed that CSs of non-western origin were less likely to respond than for platform 1. An increased non-response rate could also be caused by a decreased attention to information about PRO in Step 1, Fig. 1. The lack of utilisation of electronically administered PROs could be due to various barriers, including technical issues and usability problems with the PRO platform, concerns about data security, and the inability to use PROs due to health issues, cognitive limitations, or language barriers [14, 28].

### Implications for multipurpose use of PROs in clinical practice

When utilising PROs in a primary healthcare cancer rehabilitation setting, it becomes crucial to contemplate populations that are either not included or unresponsive to PROs. On an individual level, these populations are at risk of not undergoing a systematic health status assessment, and the benefits of preparing for their consultation with a healthcare professional by answering questions related to their health status. Previous studies have shown that systematic health status assessment via patient self-assessment reveals more symptoms than open-ended questions in consultations [11, 50]. On an aggregated level, it remains pivotal to take these populations into account while devising new service innovations based on PRO data. Furthermore, when comparing PRO data across sectors or institutions, it is imperative to consider workflows surrounding PRO and the PRO platform, as this could significantly influence both the quantity and the demographics of those who respond. Various PRO platforms are used in different settings. Both administrators and healthcare professionals should be aware that switching platforms has implications for PRO.

## Limitations

Overall, compared to other studies, the non-administration and non-response rates are low [18, 51]. Denmark is one of the most digitised countries in Europe [52], and the digital mailbox system is not comparable to many other countries. However, we believe the insights gained on how different workflows and platforms impact administration and response in clinical practice is transferable to other populations. The present study has a cross-sectional design, and we cannot establish a direct cause-effect relationship. There could be multiple causes to differences over time, and we have only looked at some of the major structural differences between the two platforms. In the rehabilitation centre, PROs are used at the initial consultation, at follow-up consultations, and at the completion consultation. We focused on the non-administration of and non-response to PRO at the initial consultation, as PRO administration is systematic and PRO as a dialogue tool is highly accepted among healthcare professionals. However, this aspect is still a work in progress for follow-up and completion consultations. Future studies should focus on adherence to PRO over time. A previous study showed that there is inequality in both referral to rehabilitation and attendance in rehabilitation [32]. Thus, the findings may not be representative of the broader cancer population. Another important limitation is the aspect of time, and effects of external factors. The two platforms were implemented sequentially, and although the implementation period for both platforms is included in the study, the study period for platform 1 includes timepoints during the Covid-19 pandemic where shutdowns and restrictions increased inequality in access to healthcare for various chronic conditions [53]. In the statistical analyses, age was grouped into 18–39 years, 40–64 years, 65–74 years, and ≥75 years. This grouping was chosen because it makes sense in our clinical setting. However, this may not be generalisable to other settings. In general, CSs under 40 years of age are defined as young adult cancer survivors and may have different rehabilitation needs than older cancer survivors [54]. In Denmark, CSs under the age of 67 are likely to be on the labour market and, as such, may have different rehabilitation needs than age-retired CSs. Over the age of 65, citizens in the municipality of Copenhagen are entitled to various home-based interventions. CSs over the age of 75 are less inclined to take up rehabilitation after referral to the centre, and when they take up rehabilitation they do not participate in as many interventions as other age groups. The audit results should only be seen as an indication of the reasons for non-administration. Reasons for non-administration were only recorded in two-thirds of audited health records, and it was not always clear whether it was the CS’s or the healthcare professional’s choice to opt out of PRO.

## Conclusion

To our knowledge, this is the first study that examines participation in PRO in a primary healthcare setting and across two different electronic PRO platforms. The present findings underpin the impact clinical workflows surrounding PROs as well as the PRO platforms used have on how many and who participates in PRO. It is therefore important to have a continuous focus on organisational health literacy responsiveness and implement changes that support the inclusion of diverse CS groups in PRO, such as, getting rid of inappropriate workflows and introducing PROs in other languages, as well as a continuous focus on how healthcare professionals communicate about PRO with CSs. A focus on continuous monitoring of the administration of and response to PROs is also important when working with PROs in clinical practice. This is to be aware of the impact of changing workflows or new platforms and the implications this might have on the individual level, and when comparing PRO answers across institutions and sectors.

### Electronic supplementary material

Below is the link to the electronic supplementary material.

Supplementary Material 1

## Data Availability

The data that support the findings of this study are available from Statistics Denmark and the Health and Care Administration of the Municipality of Copenhagen, but restrictions apply to the availability of these data, which were used under approval from the regional ethics committee for the current study, and so are not publicly available.

## List of abbreviations

PROs: Patient reported outcomes
CS: Cancer survivor
ISCED: International Standard Classification of Education
FACT-G: Functional Assessment of Cancer Therapy - General
