# Supplementary material for: Are all cancer survivors included when using electronically administered patient reported outcomes in primary healthcare cancer rehabilitation? A cross-sectional study
Source: J Patient Rep Outcomes. 2024 Jul 8;8:67. doi: 10.1186/s41687-024-00753-5 (PMC11231107; doi:10.1186/s41687-024-00753-5)
Supplement: Supplementary file 1 — Supplementary Material 1 [file 41687_2024_753_MOESM1_ESM.docx]

Supplementary table. Diagnoses for the different populations. Diagnose groups listed according to ICD10 categories for malignant neoplasms.

|  | | **Booked for needs assessment, n (%)** | | **Administered PRO, n (%)** | |
| --- | --- | --- | --- | --- | --- |
|  | | Platform 1,  Population 1a | Platform 2,  Population 2a | Platform 1,  Population 1b | Platform 2,  Population 2b |
| Diagnosis (ICD-10 codes) | |  |  |  |  |
|  | Lip, oral cavity, and pharynx (C00-C14) | 101 (5.4) | 84 (5.8) | 86 (5.3) | 64 (5.5) |
|  | Digestive organs (C15-C26) | 327 (17.5) | 225 (15.6) | 289 (17.7) | 179 (15.4) |
|  | Respiratory and intrathoracic organs (C30-C39) | 195 (10.4) | 171 (11.8) | 170 (10.4) | 141 (12.1) |
|  | Bone and articular cartilage (C40-C41) | 3 (0.2) |  | 2 (0.1) |  |
|  | Skin (C43-C44) | 28 (1.5) | 16 (1.1) | 26 (1.6) | 15 (1.3) |
|  | Mesothelial and soft tissue (C45-C49) | 18 (1.0) | 17 (1.2) | 15 (0.9) | 14 (1.2) |
|  | Breast (C50) | 600 (32.1) | 444 (30.7) | 529 (32.4) | 356 (30.6) |
|  | Female genital organs (C51-C58) | 103 (5.5) | 71 (4.9) | 94 (5.7) | 61 (5.2) |
|  | Male genital organs (C60-C63) | 111 (5.9) | 119 (8.2) | 100 (6.1) | 101 (8.7) |
|  | Urinary tract (C64-C68) | 44 (2.4) | 29 (2.0) | 41 (2.5) | 25 (2.1) |
|  | Eye, brain, and other parts of central nervous system (C69-C72) | 35 (1.9) | 30 (2.1) | 29 (1.8) | 20 (1.7) |
|  | Thyroid and other endocrine glands (C73-C75) | 5 (0.3) | 6 (0.4) | 4 (0.2) | 4 (0.3) |
|  | Ill-defined, secondary, and unspecified sites (C76-C80) | 41 (2.2) | 29 (2.0) | 37 (2.3) | 27 (2.3) |
|  | Lymphoid, haematopoietic, and related tissue (C81-C96) | 165 (8.8) | 113 (7.8) | 145 (8.9) | 92 (7.9) |
|  | Missing diagnose in referral | 92 (4.9) | 92 (6.4) | 68 (4.2) | 64 (5.5) |
